# Exploring the Gastrointestinal “Nemabiome”: Deep Amplicon Sequencing to Quantify the Species Composition of Parasitic Nematode Communities

**DOI:** 10.1371/journal.pone.0143559

**Published:** 2015-12-02

**Authors:** Russell W. Avramenko, Elizabeth M. Redman, Roy Lewis, Thomas A. Yazwinski, James D. Wasmuth, John S. Gilleard

**Affiliations:** 1 Department of Comparative Biology and Experimental Medicine, Faculty of Veterinary Medicine, University of Calgary, Calgary, Alberta, Canada; 2 Merck Animal Health, Calgary, Alberta, Canada; 3 Department of Animal Science, University of Arkansas, Fayetteville, Arkansas, United States of America; 4 Department of Ecosystem and Public Health, Faculty of Veterinary Medicine, University of Calgary, Calgary, Alberta, Canada; Universidade de Aveiro, PORTUGAL

## Abstract

Parasitic helminth infections have a considerable impact on global human health as well as animal welfare and production. Although co-infection with multiple parasite species within a host is common, there is a dearth of tools with which to study the composition of these complex parasite communities. Helminth species vary in their pathogenicity, epidemiology and drug sensitivity and the interactions that occur between co-infecting species and their hosts are poorly understood. We describe the first application of deep amplicon sequencing to study parasitic nematode communities as well as introduce the concept of the gastro-intestinal “nemabiome”. The approach is analogous to 16S rDNA deep sequencing used to explore microbial communities, but utilizes the nematode ITS-2 rDNA locus instead. Gastro-intestinal parasites of cattle were used to develop the concept, as this host has many well-defined gastro-intestinal nematode species that commonly occur as complex co-infections. Further, the availability of pure mono-parasite populations from experimentally infected cattle allowed us to prepare mock parasite communities to determine, and correct for, species representation biases in the sequence data. We demonstrate that, once these biases have been corrected, accurate relative quantitation of gastro-intestinal parasitic nematode communities in cattle fecal samples can be achieved. We have validated the accuracy of the method applied to field-samples by comparing the results of detailed morphological examination of L3 larvae populations with those of the sequencing assay. The results illustrate the insights that can be gained into the species composition of parasite communities, using grazing cattle in the mid-west USA as an example. However, both the technical approach and the concept of the ‘nemabiome’ have a wide range of potential applications in human and veterinary medicine. These include investigations of host-parasite and parasite-parasite interactions during co-infection, parasite epidemiology, parasite ecology and the response of parasite populations to both drug treatments and control programs.

## Introduction

Helminth infections have a considerable global impact on human and animal health [1–3]. Unfortunately, the methods currently used for diagnosis and surveillance have changed little in decades, and lack both sensitivity and specificity. Current methods typically involve performing fecal egg counts (FECs) to measure infection intensity, occasionally followed by conventional/real-time PCR, or fecal culture with larval morphology/morphometric analysis, to confirm species identity [4,5]. However, these approaches are low throughput, time consuming, error prone and require specialized expertise [4,6]; consequently they are only used to a limited extent. In contrast, the use of next-generation sequencing technologies is currently revolutionizing the diagnosis of bacterial and viral infections as well as opening up new areas of research including the exploration of the ‘microbiome’ [7,8]. Surprisingly, such approaches have not yet been applied to metazoan parasite communities, even though mixed infections are the norm rather than the exception in both humans and animals [9]. For example, more than ten major gastrointestinal helminth species are known to commonly co-infect cattle [10–12] and there are at least 83 distinct species of equine gastrointestinal helminths [13]. As a result, parasites often exist in complex communities and the use of next-generation deep-sequencing approaches to study parasite diversity has many applications analogous to studies of the microbiome. The interactions between these co-infecting species, are still poorly understood, and accurate quantitative assessment of the species present is critical in order to investigate these interactions. Parasitic helminths can influence host immunological responses, and have the potential to influence resistance and susceptibility to other infecting species [14]. In addition, parasites have the potential to exacerbate the deleterious effects of other co-infecting species [9,14]. This has the potential to lead to different disease outcomes than an infection with any single species [9]. Applying “microbiome” type approaches would allow the investigation of interactions between the complex assemblages of parasite species and their hosts and their consequences for transmission dynamics, immunity and parasite ecology. Such approaches also have massive potential as new tools for diagnosis and surveillance as well as for investigating the response of parasite populations to drug treatments and other control strategies.

In this paper, we describe the development of a deep sequencing assay of the ITS-2 rDNA that is analogous to 16S rDNA sequencing of bacterial communities and validate its use to accurately quantify the species composition of parasitic nematode communities present in field samples. The ITS-2 region of the rDNA cistron was chosen as the sequence target due to it having the appropriate level of species-specific variation for reliable species discrimination in Clade V nematodes [15–17]. Gastrointestinal parasitic nematodes of cattle were chosen as the system in which to develop the approach for two reasons. Firstly, co-infections with multiple, closely related Clade V nematode species, with visually identical eggs, are common in natural field infections of cattle [2]. Secondly, the availability of pure mono-specific populations derived from experimental infections allowed us to construct mock parasite communities for the determination, and correction, of species-specific biases. We show that the approach is extremely robust and quantitatively accurate when applied to field populations harvested from cattle feces and discuss how the approach is applicable to study the biodiversity of parasite populations in other domestic animal and wildlife hosts as well as in humans.

## Material and Methods

### Parasite Material

Pure populations of live third stage (L3) larvae (suspended in water) for eight of the most common cattle parasitic nematode species–*Cooperia punctata*, *Cooperia oncophora*, *Nematodirus helvetianus*, *Trichostrongylus axei*, *Trichostrongylus colubriformis*, *Haemonchus placei*, *Ostertagia ostertagi* and *Oesophagostomum radiatum*–were derived from experimentally passaged and characterized isolates. These isolates were used to provide single larvae to assess intra-species sequence diversity and to create a variety of “mock samples” of known species composition with which to develop and validate the amplicon-sequencing assay. To dispense known numbers of larvae from these isolates, the number of larvae per μL was estimated by counting larvae in at least three 10μL aliquots and then pipetting the appropriate volume from the stock flask with continuous shaking to keep the larvae as evenly suspended as possible. Larval counts will inevitably vary to some extent between aliquots, since it is not possible to produce a completely homogenous larval suspension. This will lead to some variance in the exact number of larvae of each species added to mock sample mixtures. The resulting mixtures of L3 larvae comprising the ‘mock samples’ were fixed in 70% ethanol and stored at -80°C until use.

Fecal samples were collected to provide field samples, either per rectum or as freshly voided on the pasture. Fecal samples were collected in accordance with an approved Animal Use Protocol (Animal Care Committee, Study #AC13-0157, University of Calgary), which is in accordance with the principles outlined in the current Guidelines of the Canadian Council on Animal Care. Following fecal flotation and egg counting using the Modified Wisconsin technique [18], coprocultures were set up as described in Roberts and O’ Sullivan, 1950 [19] but with the substitution of sawdust for vermiculite, and incubated at room temperature (~21°C) for 21 days. L3 larvae were harvested and stored in tap water at 6°C. The larvae harvested from individual samples were then pooled for each farm, divided into aliquots of 1000–2000 larvae, fixed in 70% ethanol and stored at -80°C until needed.

### Genomic DNA Lysate Preparations

To prepare DNA from individual larvae, sheathed L3s were picked and placed in 0.2 mL tubes containing 10 μL of lysis buffer (50 mM KCl, 10 mM Tris (pH8.3), 2.5 mM MgCl_2_, 0.45% Nonidet P-40, 0.45% Tween 20, 0.01% (w/v) gelatin). Samples were incubated at 95°C for 15 minutes, followed by incubation at -80°C for a minimum of 60 minutes. 10 μL of lysis buffer, with 120 μg/mL of proteinase K (Thermo Scientific), was added to each tube and incubated at 60°C for 98 min, followed by 20 min at 95°C to denature the proteinase K. Lysate dilutions (1:5) were prepared and stored at -20°C until use. To prepare bulk DNA lysates from larval populations, ethanol fixed larvae were washed three times by centrifugation (2500 g, 1mL lysis buffer) and re-suspended in a final volume of 100 μl lysis buffer. The larvae were heated at 95°C for 15 minutes, then frozen for at least 60 minutes at -80°C before the addition of an additional 150 μL of lysis buffer containing 120 μg/mL of proteinase K. Larvae were incubated at 60°C for 120 minutes, with shaking (750 RPM). Following incubation, proteinase K was inactivated by heating at 95°C for 20 minutes and 1:10 dilutions of lysates with molecular grade water were created and stored at -20°C until use.

### PCR Amplification and Sequencing of the rDNA ITS-2 from Single Larvae

A 311–331 bp fragment encompassing the rDNA ITS-2 sequence was PCR amplified from single larvae using the NC1 and NC2 primers described in Gasser *et al* (1993). PCR reaction conditions were 5 μL NEB 10X ThermoPol Reaction Buffer, 1 μL dNTPs (10 mM), 1 μL NC1 Primer (10 μM), 1 μL NC2 Primer (10 μM), 0.25 μL NEB Taq DNA Polymerase, 38 μL ddH_2_O and 4 μL diluted (1:10) lysate. The thermocycling parameters were 95°C for 5 minutes, followed by 35 cycles of 95°C for 30 seconds, 54°C for 30 seconds, 68°C for 1 minute followed by a final extension of 68°C for 1 minute. PCR products were purified with a MicroElute Cycle-Pure Kit (OMEGA Bio-Tek, D6293-02) and directly sequenced on both strands using Sanger sequencing with the NC1 and NC2 primers. Sequences were aligned and trimmed using Geneious version 7.1.5 created by Biomatters. Available from http://geneious.com/.

### Deep Amplicon Sequencing of the rDNA ITS-2

The overall scheme of the approach is shown in S1 Fig. Primers used to amplify the rDNA ITS-2 for deep sequencing are modifications of the NC1 and NC2 primers that are complementary to the 5.8S and 28S coding sequences respectively [20]. Adapters were added to these primers to allow the subsequent annealing of sequencing primers. Four forward (NC1Adp, NC1Adp1N, NC1Adp2N, NC1Adp3N) and four reverse primers (NC2Adp, NC2Adp1N, NC2Adp2N, NC2Adp3N) were used where Adp is the Illumina adapter sequencing tag (5’-TCGTCGGCAGCGTCAGATGTGTATAAGAGACAG-3’) and N is the number of random nucleotides included between the locus specific primer sequence and Illumina Adapter sequence to increase the diversity of generated amplicons (see S1A Table for primer sequences). Primers were offset to prevent oversaturation of the sequencing channels. The multiple forward (NC1) and reverse (NC2) primers were mixed in equal proportions, and used for PCR under the following conditions: 5 μL KAPA HiFi HotStart Fidelity Buffer (5X) (KAPA Biosystems, USA), 0.75 μL NC1+Adapter Primer (10 μM), 0.75 μL NC2+Adapter Primer (10 μM), 0.75 μL dNTPs (10 mM), 0.5 μL KAPA HiFi HotStart Polymerase (0.5 U), 13.25 μL ddH_2_O, 4 μL 1:10 dilution of worm lysate. The thermocycling parameters were 95°C for 3 minutes, followed by 25 cycles of 98°C for 20s, 62°C for 15s, 72°C for 15s, followed by a final extension of 72°C for 2 minutes. All PCR steps were carried out with best practices to reduce aerosol formation including the use of filter pipette tips, working in a biosafety cabinet, and using the easy release PCR cover Microseal ‘A’ Film (Bio-Rad MSA5001). PCR products were purified with AMPure XP Magnetic Beads (1X) (Beckman Coulter, Inc.) following the manufacturers recommended protocol. Illumina indices and P5/P7 sequencing tags were then added to the rDNA ITS-2 amplicons using limited cycle PCR amplification. Primer sequences were obtained from the Illumina Customer Sequencing Letter (September 7, 2012, Oligonucleotide sequences 2007–2012 Illumina, Inc. All rights reserved). The eight forward and 12 reverse barcoded primers were mixed to make 96 unique barcode combinations (primer sequences available in S1B and S1C Table). The following PCR conditions were used: 5μL KAPA HiFi HotStart Fidelity Buffer (5X) (KAPA Biosystems, USA), 1.25 μL Forward Primer (N501-508) (10 μM), 1.25 μL Reverse Primer (N701-712) (10 μM), 0.75 μL dNTPs (10 mM), 0.5 μL KAPA HiFi Polymerase (0.5 U), 14.25 μL H_2_O, and 2 μL of first round PCR product as template. The thermocycling parameters were 98°C for 45 seconds, followed by seven cycles of 98°C for 20s, 63°C for 20s, 72°C for two minutes. Products were purified using AMPure XP magnetic beads (1X) and a master sequencing library created by pooling ~50 ng of each purified product. The final concentration of the pooled library was assessed with the KAPA qPCR Library Quantification Kit (KAPA Biosytems, USA) following the manufacturers recommended protocol.

The prepared pooled library was run on an Illumina MiSeq Desktop Sequencer using a 500-cycle pair-end reagent kit (MiSeq Reagent Kits v2, MS-103-2003) at a concentration of 12.5 nM with the addition 25% PhiX Control v3 (Illumina, FC-110-3001). The MiSeq was set to generate only FASTQ files with no post-run analysis. The MiSeq automatically separates all sequences by samples during post-run processing by recognized indices. All protocols were carried out per Illumina’s standard MiSeq operating protocol.

### Bioinformatic and Statistical Analysis

Sequences were analyzed with our own bespoke analysis pipeline. Consensus sequences were built from raw overlapping pair-end FASTQ sequences using FLASH version 1.2.7 with default parameters [21]. BLASTN version 2.2.29+ was used to search generated sequences against a database of reference sequences generated from the sequencing of the rDNA ITS-2 sequence from the single larva derived from pure single species experimental infections [22]. A sequence identity threshold of >97% was chosen to assign each sequence to a species reference sequence to account for sequencing errors and intra-species variation in the ITS-2 region. Sequences not positively identified in this manner were searched with BLASTN against the GenBank database to obtain the best hit. Sequences that did not hit any rDNA ITS-2 sequence in Genbank were discarded as artifactual or contaminating sequences. The percentage species composition of each sample was calculated by dividing the number of raw reads per species by the total number of reads per sample. Samples with <2000 reads, were discarded, as this is indicative of a failed sample preparation. Statistics were performed in SPSS Statistics (IBM Corp. Released 2012. IBM SPSS Statistics for Macintosh, Version 21.0. Armonk, NY: IBM Corp) as required. Specific details regarding the statistical tests performed are provided in the relevant sections of the results and figure legends.

## Results

### Assessment of ITS-2 rDNA Intra- and Inter-Species Variation and the Generation of a Reference Sequence Library for Eight Parasitic Nematode Species of Cattle

**Fig 1:**
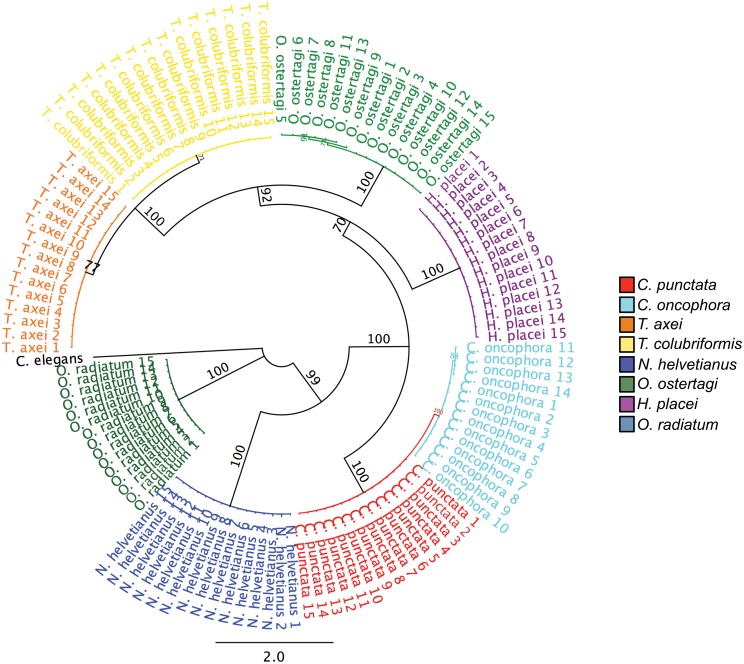
Neighbor-joining tree of ITS-2 rDNA sequences obtained from 8 different parasitic nematode species from cattle. ITS-2 rDNA amplicons were directly sequenced by Sanger sequencing from 15 individual L3 larvae obtained from experimentally passaged strains for each of eight cattle parasite species. The neighbor-joining tree (Jukes-Cantor model) was computed with 2000 bootstrap replicates and rooted on *C*. *elegans* ITS-2 (Accession: JN636100) using Geneious version 7.1.5 created by Biomatters. Available from http://geneious.com/. Bootstrap values >70 are shown, values >80 are considered to be significant.

The ITS-2 rDNA was amplified and sequenced from 15 individual L3 larvae taken from the characterized laboratory passaged isolates of *C*. *punctata*, *C*. *oncophora*, *N*. *helvetianus*, *T*. *axei*, *T*. *colubriformis*, *H*. *placei*, *O*. *ostertagi* and *O*. *radiatum* to assess intra- and inter-species variation. Alignment of the sequences revealed differences in inter-species sequence identity ranging from 49.0% to 98.8% (S2A Table). In contrast, the intra-species sequence identity varied from 99.2% to 100% (S2A Table). A phylogenetic analysis demonstrates distinct clustering between species (Fig 1). The two most closely related species, *Cooperia oncophora* and *Cooperia punctata*, could still be reliably differentiated by virtue of three fixed single nucleotide polymorphisms (SNPs) (S3B Table), as could the next two closely related species *Trichostrongylus axei* and *Trichostrongylus colubriformis* by virtue of nine fixed SNPs (S3C Table). Generated sequences were compared to all available ITS-2 sequences in GenBank for each species to account for any additional documented diversity and no additional fixed species-specific SNPs were identified with this analysis (S3A Table). With these additional sequences, intra-species sequence identity decreased to 96.8–100% (S2B Table). Consensus sequences for each species were created from the generated sequences and used as the reference sequence library for the subsequent work (S3A Table). All new generated sequences have been submitted to GenBank (Accession Numbers: KP150445-KP150563).

### Assessment of Sequence Representation Bias for the Different Parasite Species and the Determination of ‘Correction Factors’

**Fig 2:**
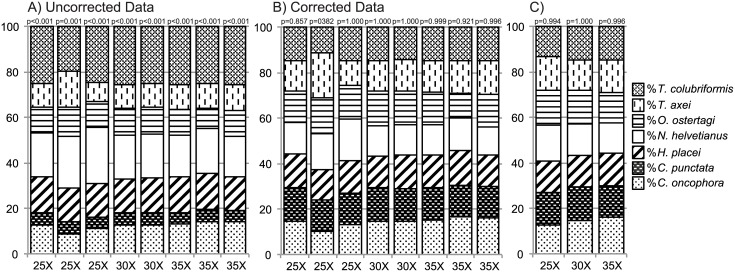
Assessment of sequence representation bias and the determination of correction factors for the amplicon sequencing assay. A DNA lysate was prepared from a pool of 140 L3 larvae comprising 20 individual L3 larvae from each of the following species; *N*. *helvetianus*, *C*. *oncophora*, *T*. *axei*, *T*. *colubriformis*, *C*. *punctata*, *O*. *ostertagi* and *H*. *placei*. The sequencing assay was applied to this lysate eight times; three, two and three times at 25, 30 and 35 cycles of amplification respectively (as denoted on the x-axis of panels A and B). **2A**. The chart shows the species proportions as determined by the actual number of ITS-2 rDNA sequences generated by the amplicon sequencing assay. The p-values above each column indicate whether the species proportions, as determined by the uncorrected numbers of ITS-2 rDNA sequences generated, are statistically different from the actual proportions of larvae in the pool (non-parametric Chi-square test with significant differences between expected and observed results (p≤0.001) for all replicates). **2B**. The chart shows the same data as in panel 2A but following the application of a correction factor for each species. The correction factor was calculated for each species by dividing the true proportion of that species in the pool (based on counted larvae) by the mean proportion of Illumina sequences generated from the eight replicates (correction factor = %Actual / %Observed). The correction factors were: *N*. *helvetianus* = 0.68871028, *C*. *onchophora* = 1.183576925, *T*. *axei* = 1.296071418, *T*. *colubriformis* = 0.584257283, *C*. *punctata* = 2.779407405, *O*. *ostertagi* = 1.294841767 and *H*. *placei* = 0.919208106. Their application removed the significant variation (p>0.05). The p-values above each column indicate whether the species proportions, as determined by the corrected numbers of ITS-2 rDNA sequences generated, are statistically different from the actual proportions of larvae in the pool (non-parametric Chi-square test). **2C**. Replicates were grouped and averaged based on cycles of amplification. A one way ANOVA was performed to detect if number of cycles of amplification affected the proportional representation of each species with non-significant results (*N*. *helvetianus*, F = 1.200, p = 0.375; *C*. *oncophora*, F = 3.035, p = 0.137; *T*. *axei*, F = 0.098, p = 0.908; *T*. *colubriformis*, F = 0.752, p = 0.518; *C*. *punctata*, F = 2.683, p = 0.162; *O*. *ostertagi*, F = 2.501, p = 0.177; *H*. *placei*, F = 1.672, p = 0.278)

To assess quantitative accuracy and determine any sequence representation bias for the different parasite species, a mock sample was prepared by individually picking 20 L3 larvae of each of seven species into a single pool (total of 140 larvae) (*O*. *radiatum* was not included as it was not available at the time of preparation). The amplicon sequencing assay was then performed on this pool eight independent times. The number of cycles for the first round PCR was varied to assess its impact on the sequence representation of each species (Fig 2). Each of the species were either significantly underrepresented or overrepresented by the actual number of sequences generated relative to their true proportions in the mixture (Fig 2A). However, these biases were very consistent between replicates and did not change drastically as the number of amplification cycles was varied (Fig 2A). This consistency allowed a “correction factor” to be calculated and applied to the data to reduce the observed sequence representation biases (Fig 2B). The application of the correction factor removed the significant difference between observed and expected values (p>0.05) (Fig 2B). Calculating the mean values based on cycles of amplification removed further deviation from expected values, indicating that level of amplification does not influence the proportional representation of species (Fig 2C). Additionally, this demonstrates that performing replicates of the same samples should minimize any differences in representation produced by the assay.

### Validation of the Amplicon-Sequencing Assay Using Artificially Prepared Mixtures of Known Species Composition

**Fig 3:**
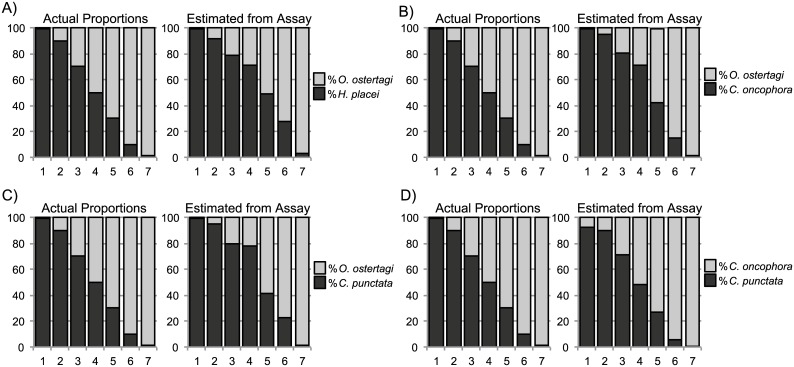
The determination of species proportions of a series of pairwise parasite species combinations using the amplicon sequencing assay. DNA lysates were prepared from mock pools of 2000 L3 larvae comprising varying proportions of two different nematode species and the amplicon sequencing assay applied. Panel 3A, *O*. *ostertagi* and *H*. *placei*; Panel 3B, *C*. *oncophora* and *O*. *ostertagi*; Panel 3C, *O*. *ostertagi* and *C*. *punctata*; Panel 3D, *C*. *oncophora* and *C*. *punctata*. The left hand chart of each pair shows the expected results based on the known numbers of larvae added to each pool and the right hand chart of each pair shows the results of the amplicon sequencing assay (after the application of the appropriate correction factors). The ratios by which the two species of each pair vary from left to right on each chart are as follows: Column 1, 99:1; Column 2 90:10; Column 3; 70:30, Column 4, 50:50; Column 5; 30:70, Column 6; 10:90 and Column 7; 1:99.

First we tested the ability of the assay to accurately determine the relative species proportions of a series of pairwise combinations of parasite species. A series of pools of ~2000 larvae were created, each consisting of differing proportions of two nematode species in the following ratios: 99:1, 90:10, 70:30, 50:50, 30:70, 10:90 and 1:99. This was performed for the following species pairs: *C*. *oncophora*/*O*. *ostertagi*, *C*. *punctata*/*O*. *ostertagi*, *O*. *ostertagi*/*H*. *placei* and *C*. *oncophora*/*C*. *punctata*. These pairs were selected based on their likely co-occurrence in cattle herds. The relative sequence proportions accurately reflected the actual species proportions of the larvae in the mixture (Fig 3).

**Fig 4:**
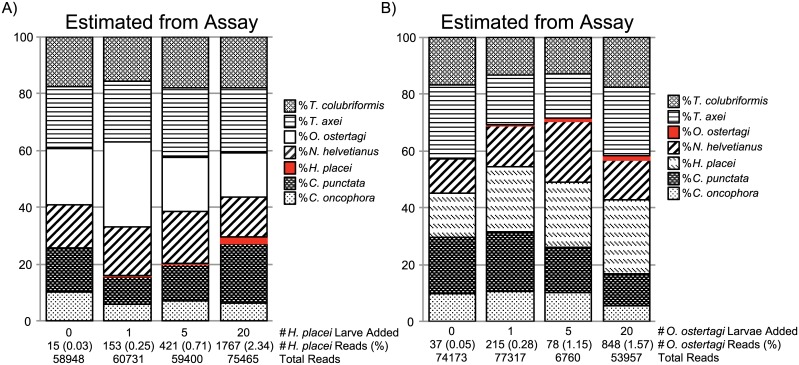
Detection threshold of the amplicon sequencing assay. Separate mock pools of ~2000 larvae were created consisting of equal proportions of 6 parasite species. The seventh species was then added in increasing numbers of 0, 1, 5 and 20 larvae respectively. The charts show the species proportions estimated from the results of the amplicon sequencing assay (following application of the appropriate correction factor). In each case, the Y-axis shows the percentage proportions of each species and the X-axis indicates the number of *H*. *placei* or *O*. *ostertagi* larvae added as well as the numbers of reads assigned to *H*. *placei* and *O*. *ostertagi* for **Fig 4A** and **4B** respectively. **Fig 4A**. Results when *H*. *placei* was added in increasing numbers to a pool comprising an equal number of *N*. *helvetianus*, *C*. *oncophora*, *T*. *axei*, *T*. *colubriformis*, *C*. *punctata* and *O*. *ostertagi*. **Fig 4B**. Results when *O*. *ostertagi* was added in increasing numbers to a pool comprising an equal number of shows *N*. *helvetianus*, *C*. *oncophora*, *T*. *axei*, *C*. *punctata*, *T*. *colubriformis* and *H*. *placei*.

Next, we tested the detection threshold of the assay for each of the species when present at low levels in mixed populations. Separate pools of ~2000 larvae were created consisting of equal proportions of 6 parasite species. The remaining absent species was then added in increasing numbers of 0, 1, 5 and 20 larvae respectively. Such a sample series was prepared with each of the main seven species being absent from the mixture but then being subsequently added, resulting in 28 pools total. The sequencing assay was then applied to these pools using the standard number of amplification cycles (25X) and applying the correction factor described in the previous section (Fig 4A and 4B). One challenge to the sensitivity of any deep sequencing assay is the detection of the trace amplicon contamination that inevitably occurs during sample handling subsequent to the initial PCR amplification. Minor sequence contamination does not affect the interpretation of the relative species proportions since the trace contamination was always less than the number of sequences corresponding to those generated from a single parasite larva in a sample. Consequently, contaminating sequences can be distinguished from genuine data. Further, the very small changes in species proportions associated with trace contamination do not change the biological interpretation of the data for the applications we have discussed. In the example shown in Fig 4, 15 out of 58,948 and 37 out of 74,173 reads 15 reads were assigned to *H*. *placei* and *O*. *ostertagi* respectively in pools where those species were absent (as denoted below the X-axis on the first row of each chart). However, read counts never exceeded 0.05% of the total read count for a species when absent from a pool, whereas the addition of a single larvae increased the read counts above that level. In the example in Fig 4, the addition of a single *H*. *placei* or *O*. *ostertagi* larva to pools resulted in 153 out of 60,731 reads and 215 out of 77,317 reads respectively (as denoted below the X-axis of the second column of each chart). Hence, the method is capable of reliably detecting the presence of a single larva in a pool of 2000 above trace contamination. Further, the sequence proportions increased as predicted as more larvae were added to each pool (Fig 4). Similar results were obtained for the other species assessed.

### Repeatability of Amplicon Sequencing Applied to Field Samples

**Fig 5:**
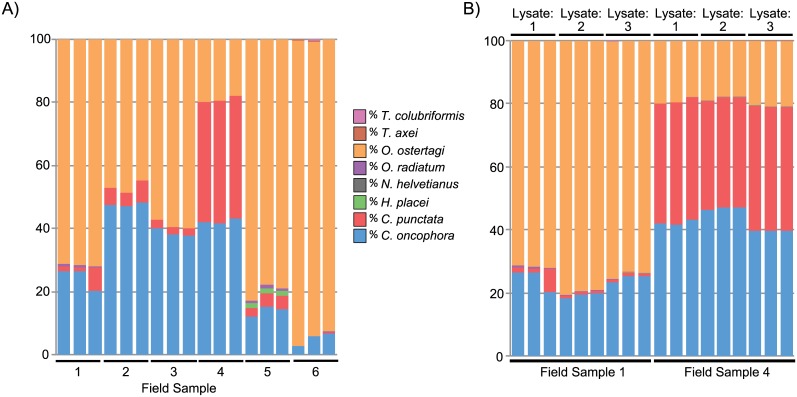
Repeatability of the amplicon sequencing assay when applied to field samples. The amplicon sequencing assay was applied to DNA lysates made from populations of L3 larvae collected from field samples from six Canadian cattle farms (Field samples 1–6). **Fig 5A**: The results of three technical replicates of the amplicon sequencing assay applied to the same single DNA lysate made from larval populations of each farm. Each DNA lysate was made from 300–2000 larvae (Field sample 1 = 1000 L3; Field sample 2 = 400 L3; Field sample 3 = 1000 L3; Field sample 4 = 2000 L3; Field sample 5 = 300 L3 and Field sample 6 = 2000 L3. **Fig 5B**: The results of three technical replicates of the amplicon sequencing assay applied to three independent DNA lysates made from separate batches of 1,000 and 2,000 larvae from field samples 1 and 4 respectively. The Y-axis of both charts shows the percentage species proportions as determined by the amplicon sequencing assay (25 cycles of amplification and following application of the appropriate correction factor). The field sample number is indicated on the X-axis.

Having developed and validated the assay on artificially created pools of larvae, we then tested the assay when applied to field samples. L3 larvae were harvested from individual coprocultures of calf fecal samples collected from six different Canadian cow-calf herds (twenty calves per farm). The larvae from each farm were separately pooled and a DNA lysate prepared for each pooled sample. The deep sequencing assay was then applied three separate times to each of the six resulting DNA lysates. There was a high level of reproducibility for the three technical replicates of each DNA lysate, with the range for each species being typically within 1–2% (maximum of 6%) between replicates (standard deviations; 0.005–3.5%) (Fig 5A). The major species detected were *O*. *ostertagi*, *C*. *oncophora* and *C*. *punctata*, which were present at a range of different proportions on each farm. In addition, the assay repeatedly detected two additional species, *O*. *radiatum* and *H*. *placei*, at very low proportions (~1%) on several farms.

The samples from two farms (Farm 1 and 4) were selected for further analysis. In these cases, three separate aliquots of harvested larvae were lysed to produce three independent DNA lysates per farm (Fig 5B). The amplicon sequencing assay was applied to each DNA lysate in triplicate. The repeatability, both across the three technical replicates of each lysate and across the three lysates from each farm, was very high. The range of percentage proportions for each species being <2% between replicates and <9% between lysates with the exception of the third technical replicate of lysate 1 of field sample 1 which showed an over representation of *C*. *punctata* (Fig 5B).

### Comparison of the Amplicon Sequencing with Visual Morphological Identification of Larval Populations from Field Samples

**Fig 6:**
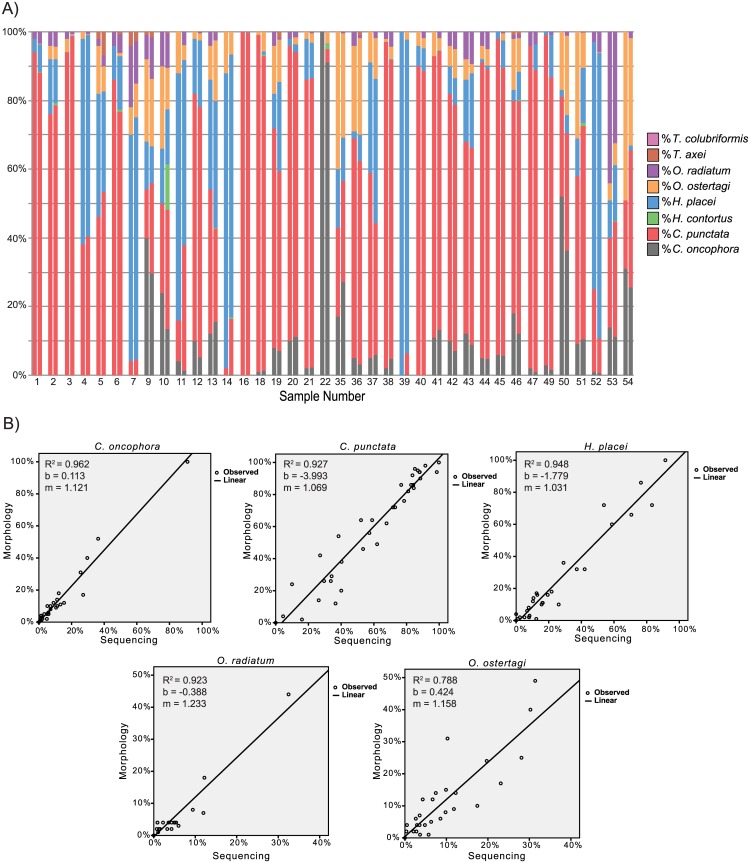
Comparison of the amplicon sequencing assay with visual morphological identification of larval populations from field samples. Fecal samples were collected from 39 individual calves entering feedlots from pasture at different locations in Oklahoma, Arkansas and Nebraska. 100 L3 larvae harvested from each coproculture were fixed and identified to the species level on the basis of morphological features on microscopy. The amplicon sequencing assay was applied to several hundred to several thousand L3 larvae from each sample. **Fig 6A**. Two bars are shown for each sample on the chart. The left bar of each sample shows the species proportions as determined by larval morphology. The right bar of each sample shows the proportions of each species as estimated by the amplicon sequencing assay (values shown are after application of the appropriate correction factor). The Y-axis shows the percentage proportions of each species as determined by morphology or the sequencing assay respectively. **Fig 6B**. Linear regression analysis was performed to assess the correlation between the percentage composition of each parasite species in each of 39 field samples as determined by the amplicon sequencing assay and morphological identification. The linear regression plots were produced using SPSS Statistics (IBM Corp. Released 2012. IBM SPSS Statistics for Macintosh, Version 21.0. Armonk, NY: IBM Corp), by plotting the percentage representation of each species from the morphological data (Y-axis) against the percentage representation of each species from the amplicon sequencing data (X-axis) for each sample. The values for R^2^ (coefficient of determination), b (y-intercept) and m (slope) are shown adjacent to each plot. If values are the same the y-intercept (b) will be zero and the slope (m) will be 1.

Fecal samples were collected from 39 calves upon feedlot entry that were derived from different pastures in Oklahoma, Arkansas and Nebraska. For each of the 39 samples, an aliquot of 100 L3s derived from coprocultures were visually examined by microscopy and identified based on their morphological features [4,23]. The sequencing assay was then applied to the DNA lysates prepared from the remaining larvae (several hundred to several thousand L3s) from each sample and the species proportions determined based on the mean of two independent replicate assays and the relevant correction factors applied. There was a high level of agreement between the parasite species proportions as determined by the sequencing assay compared with those determined on the basis of larval morphology (Fig 6A). Linear regression analysis was performed to assess the correlation between the percentage composition in each sample for each species as determined by the sequencing assay and morphological identification (Fig 6B). There was a high degree of correlation between the results of the two methods for all species included in the analysis (where R^2^ is the coefficient of determination, b is the y intercept and m is the slope): *C*. *oncophora*, R^2^ = 0.962, b = 0.113, m = 1.121; *C*. *punctata*, R^2^ = 0.927, b = -3.993, m = 1.069; *H*. *placei*, R^2^ = 0.948, b = -1.799, m = 1.032; *O*. *radiatum* R^2^ = 0.923, b = -0.388, m = 1.233; *O*. *ostertagi*, R^2^ = 0.788, b = 0.424, m = 1.158. Regression analysis was not performed for *H*. *contortus* and *T*. *colubriformis* since they were not detected in the morphological analysis or for *T*. *axei*, as there were insufficient data points due its rare occurrence in the samples. For Sample 10, *H*. *contortus* was detected by the sequence-based assay but not by the morphological examination. This is likely due to the difficulty of distinguishing between *H*. *contortus* and *H*. *placei* on morphological criteria when present in cattle fecal samples (T. Yazwinski, per comm). This is supported by the observation that *H*. *contortus* was known to be present in other cattle samples from the same location (T. Yazwinski, per comm).

## Discussion

### Interrogating the “Nemabiome”: Deep Amplicon Sequencing Has Wide Applications to Study Parasitic Nematode Communities in Many Host Species

We have developed a new approach, analogous to 16S rDNA sequencing used in microbiome studies, which can be used to investigate the species composition of parasitic nematode communities present in mammalian hosts. The ability to accurately quantify the parasite species composition in fecal samples from individuals, groups and geographical regions, as well as to monitor changes over time or following drug treatments, has many potential applications in both human and veterinary medicine. Co-infection with multiple parasite species is common and has been shown to affect immunological responses and infection dynamics [14,24]. However, despite this, such relationships are still largely unexplored. In domestic animals, gastrointestinal nematode communities can be extremely complex and can be conceptually described as the gastrointestinal “nemabiome”. For example, up to 25 different nematode species infect cattle in North America with at least 10 of these being of economic importance [12]. In horses, there are at least 83 distinct species of gastrointestinal helminths, with a single host often being co-infected with over a dozen species [13]. Parasite co-infections are common in humans too, with individuals in the developing world often being infected with multiple gastrointestinal parasite species [1]. Although these metazoan parasite communities are of lower complexity than bacterial communities, the potential for interspecies interactions is significant and as of yet unexplored. Deep sequencing methodologies, such as the one described here, will provide a powerful approach with which to investigate metazoan parasitism in a manner analogous to current trends in microbiome research that are revolutionizing the study of bacterial communities.

There are also important immediate clinical diagnostic applications of this deep sequencing approach. In cattle, sheep and horses, the majority of the most important gastrointestinal nematode species belong to the order Strongylida and have visually identical eggs. However, many of these species vary dramatically in pathogenicity and drug sensitivity. For example, *Haemonchus* species are extremely pathogenic in sheep due to their blood feeding nature, whereas *Cooperia* species are much less damaging [12]. In cattle, *Ostertagia ostertagi* is generally susceptible to ivermectin whereas *Cooperia* species are increasingly resistant [2,12]. In humans, the eggs of the hookworm species *Necator americanus* and *Ancylostoma duodenale* are visually indistinguishable. Hence, the ability to accurately quantify the relative proportions of these species in fecal samples provides diagnostic information that is immediately applicable to either control or, in the case of human parasites, surveillance. Although a variety of molecular assays have been developed to detect these species in the past, they have been generally based on conventional or real-time PCR technology [16,17,25,26]. However, these assays only detect species that have been previously included and optimized in the assay design and often have problems with biased and variable amplification and can be difficult to replicate between labs [16,25,27]. Hence, they have significant limitations regarding reproducibility, accuracy and scalability. Deep amplicon sequencing assays, such as the one described here, not only have the potential to provide more accurate and reliable quantification of parasite community species diversity but can also detect previously unanticipated parasite species. Additionally, while the assay provides relative quantitation of species prevalence, combining the obtained species quantitation data with fecal egg count data can provide an infection intensity for each species. The use of indexed primers enables large numbers of samples to be pooled and sequenced in a single Illumina sequencing lane, reducing materials and labour costs, and making the approach suitable for high throughput analysis. By multiplexing the primer combinations outlined in this paper it is possible to sequence 96 samples at once on a single MiSeq flow cell. However, additional barcodes can be generated to increase the number of samples that can be simultaneously sequenced, thereby saving time and reducing costs. Increased multiplexing, together with the decreasing cost and increasing capacity of massively parallel sequencing platforms, is likely to make this approach increasingly economic and practical for many routine research and diagnostic applications.

### Defining and Correcting for Species Representation Bias

One major technical advantage of working with parasitic nematode communities, compared to microbial communities, is that it is easier to explore and correct for species representation biases in the sequence data. One reason we have developed the approach using cattle gastro-intestinal parasites is that we were able to obtain pure mono-specific cultures from experimental infections. This allowed us to make a variety of mock communities of L3 larvae in order to define, and then correct for, species representation biases. This is rarely undertaken in microbiome studies due to the high complexity of bacterial communities and the inability to culture many of the species that are present [28,29]. In contrast, the cattle gastrointestinal parasite system has provided us with a unique opportunity to assess the biases inherent with this type of work and be able to accurately correct for these biases and then validate the assay. As evidenced in Fig 2, there are significant inherent biases present in species representation. There are a variety of possible reasons for this including: (i) Species-specific differences in DNA abundance due to differences in L3 larval size and number of cells or nuclei. (ii) Differences in the efficiency of DNA lysis between species. (iii) Species-specific differences in copy number of the ITS-2 rDNA target. (iv) Sequence polymorphism in primer binding sites; unlikely in this case, as the binding sites are conserved for all Clade V nematodes assessed to date. (v) Differences in amplification efficiency due to species-specific sequence variation in the amplicon. Of these possibilities, copy number variation (CNV) of the rDNA cistron is likely to be a major contributor since it is present as a multi-copy tandem repeat and copy number variation has been suggested for free-living nematodes [30,31]. Differences in amplification efficiency due to sequence variation might be expected since amplification efficiency depends heavily on the GC content and secondary structure of the sequence target and there is marked sequence variation in the ITS-2 rDNA between some of the species in this study [32,33]. However, if amplification efficiency is a major factor, biases would be predicted to become more pronounced with increased amplification cycles, which was not the case in this study (Fig 2C).

Although it will be interesting to define the precise sources of bias, it is not essential in order to accurately compensate for them and validate the assay. This is because the biases are very consistent between replicated assays, with little variance between repeated samples from the same parasite DNA preparation, or for biological replicates obtained from the same parasite community (aliquoted and lysed independently) (Figs 2 and 5). Consequently, as long as a standardized protocol is used, species representation biases can be corrected for. We have calculated “correction” factors for each of the major cattle gastrointestinal parasite species and these perform well across a wide range of different species mixtures. In Fig 6, it can be seen that when the correction factors are applied, the assay accurately reflects the species proportions, as determined by independent morphological assessment of larval populations, across a large number of field samples of varying species composition. The only differences observed between the results of the sequencing assay and the morphological examinations are in those species that are present at low frequency. This is unsurprising since, due to the labor-intensive nature of morphological identification of individual larvae, only 100 larvae per sample were morphologically examined compared to between several hundred and several thousand for the sequencing assay. Hence when the species are present at just a few percent (or less), the morphological data is likely to be less accurate.

### Application of Deep Amplicon Sequencing to Explore the Cattle Gastro-Intestinal “Nemabiome”

There are a large number of parasitic nematode species that infect the bovine gastrointestinal tract and the species composition of the parasite communities present in individual hosts is affected by a large number of factors including climatic zone, geographical location, time of year, co-grazing with other domestic animal species or wildlife and a variety of other husbandry practices and control measures including anthelmintic drug dosing. Many of the different parasite species vary significantly in terms of their pathogenicity, production impact, epidemiology and drug sensitivity and so defining the species composition is extremely important from a management perspective [12,34]. Species identity is typically determined by culturing the feces, to allow eggs to hatch and larvae to subsequently develop to the L3 stage. A representative number of individual L3 larvae harvested from the sample are then morphologically identified [23]. This is not only extremely time consuming, but is a highly specialized task and prone to error depending on the expertise of the individual undertaking the procedure. And while cheap in terms of reagents required, morphological approaches can be very expensive as a result of highly-skilled labour costs. As mentioned previously, both conventional and real time PCR assays have been developed for several of the cattle parasite species but these are only partially quantitative and are far from comprehensive in terms of the range of species present in many parasitic nematode communities [16,17,25,35]. In contrast, the deep amplicon sequencing approach described here is not only comprehensive in its ability to detect all strongylid nematode species in a sample but is also suitable for high throughput analysis. In principal, the species composition of hundreds of samples at a time can be quantitatively determined by a single, minimally trained operator. Additionally, these types of assays have the potential to be automated, greatly reducing hands on time for paid personal. This opens up new possibilities to study the epidemiology, ecology and pathobiology of these parasites as well as providing a surveillance tool of a different scale compared with conventional approaches.

The deep amplicon sequencing approach was intentionally developed using L3 larvae (harvested following in vitro culture of fecal samples) since these are at an arrested stage of development; all strongylid parasites present in cattle fecal samples (with the exception of *N*. *helvetianus*) reach the arrested L3 stage by 10 days of fecal culture at 21°C [36,37]. This allowed us to apply a standard protocol to samples, which, by including a 21-day fecal incubation step, minimized any sample-to-sample variation due to differences in parasite development, and allowed all species to reach the L3 stage. In contrast, attempting to apply this approach to parasite eggs harvested from fresh fecal samples is likely to introduce additional sample-to-sample variation. For example, a fecal sample that is 6 hours old will contain parasites at different stages of development than one that is 12 or 24 hours old. Given that the number of cells, and thus DNA content, will change as the parasites develop, and that the different parasite species develop at different rates, this would possibly introduce additional biases that would be difficult to correct for when samples are being collected from the field. Consequently, although it would be more convenient to use fresh fecal samples, particularly for clinical diagnostic work, we believe the use of harvested L3 larvae from cultured feces is likely to be the most repeatable and accurate quantitation of the parasite communities in the sample. However, if this approach were developed for diagnostic purposes, the use of eggs would provide a more rapid assay. The application of the approach to eggs in fresh feces will be an important area for future investigation.

The sequencing assay is also an improvement on the traditional microscopy-based approaches as certain L3 larvae are difficult to identify to the species level by morphological or morphometric analysis alone [23]. For example, in field sample 10 (Fig 6), the sequencing assay detected the presence of *H*. *contortus* at 13.1% in the sample. This species, which is predominantly a parasite of small ruminants rather than cattle, was not detected by morphological analysis due to these larvae originally being categorized as *H*. *placei* due to overlap in the morphometric criteria between the two species [23]. *H*. *contortus* was actually detected at low frequency in a number of the samples (2, 6, 13, 14, 18, 22, 46, 51) by the sequencing assay but not the morphological examination. This demonstrates that an additional advantage of the approach is the detection of rarer species in fecal samples (Fig 6A).

The data generated from the set of field samples used to validate the method illustrates the valuable information that can be provided by this approach (Fig 6). For example, the data reveals the predominance of *C*. *punctata* in fecal samples from grazing cattle entering feedlots sourced from pastures located in Nebraska, Oklahoma and Arkansas. Traditionally *C*. *oncophora* and *O*. *ostertagi* have been considered to be the most prevalent species in US cattle but it has been suggested that *C*. *punctata* has become much more common in recent years perhaps as a result of selection by routine macrocyclic lactone use [12]. This is of importance because *C*. *punctata* is significantly more pathogenic than *C*. *oncophora* [12]. However, there is a lack of published data to support this, due to the huge undertaking needed to accurately quantify species proportions either from fecal samples or on post-mortem material. The data presented here provides some objective evidence of the currently high prevalence of *C*. *punctata* in grazing cattle in the southern mid-west USA. The ability to apply this approach to larger numbers of samples from across North America will enable this hypothesis to be further explored. This data also reveals striking differences in the species composition between some of the different cattle herds sampled. For example, samples 14, 39 and 52 (Fig 6A) are predominantly composed of *H*. *placei*, which is an extremely pathogenic nematode species due to its blood feeding behavior. In contrast, sample 22 is composed almost entirely of *C*. *oncophora*, which is a very mild pathogen. Hence, these different infections will have very different animal health and production impacts.

## Conclusion

Consideration of parasite interactions during co-infection and their effect on immunity, pathogenesis, epidemiology and ecology is a hugely neglected area of research. In this paper, we provide the first application of deep amplicon sequencing to quantify the species composition of metazoan parasite communities and illustrate its reliability and utility when applied to field populations. This new methodology has many potential diagnostic and research applications in humans, domestic animals and wildlife. This paper also introduces the concept of the gastro-intestinal “nemabiome” to describe the parasitic nematode community present in a single host that can now be defined and studied in a way not previously possible.

## Supporting Information

S1 Dataset File includes all relevant data used to generate the Figs 2, 3, 4, 5 and 6. The data included is in the form of number of reads assigned to each species.
(XLSX)

S1 Fig Schematic representation of the preparation of the Illumina sequencing Library. In the initial PCR amplification, overhanging primers are used to amplify the ITS-2 rDNA region to generate a fragment that includes the ITS-2 amplicon as well as the Rd1 and Rd2 specific primer regions. The Rd1 and Rd2 regions provide the target sites for the primers used for sequencing the fragment. 0–3 random nucleotides (‘N’s) are inserted between Rd1/Rd2 sequences and the binding site of the primers to offset the reading frame, thereby increasing diversity when the amplicons are sequenced to prevent oversaturation of the MiSeq sequencing channels. A second, limited-cycle PCR reaction is then performed using overhanging primers that bind to the Rd1 and Rd2 tags of the amplicon to append indices as well as the P5 and P7 regions required to bind to the Illumina flow cell.
(EPS)

S1 Table PCR primer sequences for library preparation. **S1A)** Primer sequences for ITS-2 Amplification. NC1/NC2 Primer sequence bolded, N’s are underlined. **S1B)** Forward Indexed Primers. Index sequence bolded **S1C)** Reverse Indexed Primers. Index sequence bolded.
(EPS)

S2 Table Inter and Intra species variation of ITS-2 Sequence Identity. **A)** Consensus sequences were generated from 15 (14 for *Cooperia oncohphora*) rDNA ITS-2 sequences obtained from individual larvae for each of 8 species. Sequences were analyzed using Geneious version 7.1.5, and trimmed to remove the 5.8S and 28S regions leaving only the ITS-2 locus. Sequences were aligned with MUSCLE Alignment [38] using default parameters. The percent sequence identity between each consensus sequence is listed. The variation of intraspecies sequence identity for each species is indicated directly underneath each species name. **S2B)** All ITS-2 sequences currently available in GenBank were compiled for each species, and consensus sequences generated. Sequences were analyzed using Geneious version 7.1.5, and trimmed to remove the 5.8S and 28S regions leaving only the ITS-2 locus. Sequences were aligned with MUSCLE Alignment [38] using default parameters. ITS-2 locus. The percent sequence identity between each consensus sequence is listed. The variation of intraspecies sequence identity for each species in noted underneath each species name.
(EPS)

S3 Table ITS-2 consensus sequences and fixed SNP differences between parasite species. **S3A)** ITS-2 sequences were generated from 15 (14 for *Cooperia oncohphora*) rDNA ITS-2 sequences obtained from individual larvae for each of 8 species as well as all ITS-2 sequences available from Genbank at time of publication. Sequences were analyzed using Geneious version 7.1.5, and trimmed to remove the 5.8S and 28S regions leaving only the ITS-2 locus. Sequences were aligned with MUSCLE Alignment using default parameters [38]. Consensus sequences were generated using a 95% threshold. Variable positions for each species are underlined and bolded. Fixed single nucleotide polymorphisms (SNPs) between *Cooperia spp*. (**S3B**) and *Trichostronglyus spp*. (**S3C**) are identified.
(EPS)
